# Identification of *Mycobacterium leprae* and *Mycobacterium lepromatosis* Cases with Broad-Range Molecular Assays at a Single Large Reference Laboratory

**DOI:** 10.4269/ajtmh.25-0379

**Published:** 2026-04-21

**Authors:** Gregory S. Olson, Khalil Deveaux, Diana Chiller, Dhruba SenGupta, Brad T. Cookson, Jason D. Simmons, Joshua A. Lieberman

**Affiliations:** ^1^ Department of Laboratory Medicine and Pathology, University of Washington, Seattle, Washington, USA;; ^2^ School of Medicine, Tulane University, New Orleans, Louisiana, USA;; ^3^ School of Public Health and Tropical Medicine, Tulane University, New Orleans, Louisiana, USA;; ^4^ Department of Medicine, University of Washington, Seattle, Washington, USA

## Abstract

Definitively diagnosing Hansen’s disease (HD) is challenging, but molecular methods can provide species identification of the causative mycobacteria (*Mycobacterium leprae* and *Mycobacterium lepromatosis*). Most studies have investigated targeted assays, and the performance of broad-range molecular approaches remains unknown. This study evaluated a multilocus (*hsp65*, 16S ribosomal RNA, and *rpoB*) broad-range polymerase chain reaction (PCR) assay in detecting HD-associated mycobacteria at a single large reference laboratory in the United States from 2009 to 2024. We identified 148 positive samples from 137 patients submitted from 26 states. *Mycobacterium lepromatosis* was identified in 8 of 137 patients (5.8%), and *M. leprae* was identified in the rest. Although most samples were skin biopsies (∼90%), 10 samples (7.9%) represented atypical locations, including bone marrow and liver. Half of all samples were identified by only a single target (*n* = 72/146 samples, 49.3%), and *rpoB* was the most likely to be positive (*n* = 123/146 samples, 84.2%). Overall, broad-range PCR successfully identified HD-associated mycobacteria, including *M. lepromatosis*, from skin and atypical anatomic sites, and it should be considered more frequently in the evaluation of suspected cases.

## INTRODUCTION

Hansen’s disease (HD) or leprosy is a chronic bacterial infection that has been around for millennia, making it one of the oldest known human infections.^1^ Hansen’s disease remains ubiquitous in tropical countries, especially in underdeveloped and developing regions, with ∼200,000 new cases per year globally.^2^ In the United States, there are ∼200 cases reported annually, although this is likely an underestimate because of underdiagnosis and inconsistent reporting to local health jurisdictions.^3^^,^^4^ Hansen’s disease mainly affects the skin and nerves, but it can involve other tissues, including oral mucosa,^5^ upper respiratory tract,^6^ eyes,^7^ and testes.^8^ The clinical manifestations reflect diverse host immunologic responses that result in the dichotomous outcomes of paucibacillary or multibacillary disease in the WHO classification system.

Two species of acid-fast, Gram-positive obligate intracellular bacilli of the family Mycobacteriaceae cause HD: *Mycobacterium leprae*, the predominate species, and *Mycobacterium lepromatosis*, which was originally reported to be associated with the more severe diffuse lepromatous leprosy disease and cutaneous vasculitis termed Lucio reaction.^9^ Whether *M. lepromatosis* tends to cause more severe disease remains controversial. Discovered only in 2008,^10^ *M. lepromatosis* has been found in a large proportion of cases in Mexico and Central/South America, including ∼45% of cases in Mexico or in Mexican-born persons in a recent meta-analysis.^11^ Two series investigating samples from the United States showed that the combined proportion owing to *M. lepromatosis* was ∼16% (*n* = 19/120 HD patients), including a small percentage of dual infections,^9^^,^^11^^,^^12^ but the true proportion of HD caused by *M. lepromatosis* in the United States remains understudied.

Although prompt treatment of HD is required to prevent permanent damage and transmission, early diagnosis remains challenging, in part because of the inability to culture the causative mycobacteria. Hansen’s disease diagnosis relies on clinical examination, which may identify anesthetic plaques, diffuse lepromatous nodules, thickened peripheral nerves, and/or neuropathy. In multibacillary disease, slit-skin smears^2^^,^^13^ and/or histopathologic examination of a skin biopsy may identify causative mycobacteria with a classical Acid Fast Bacillus (AFB) stain (e.g., Ziehl–Neelsen), which is suboptimal for the evaluation of HD-causing mycobacteria, or a modified AFB stain (e.g., Fite stain, which is optimized for the less acid-fast *M. leprae*). Molecular evaluation of biopsy tissue using quantitative polymerase chain reaction (PCR) is confirmatory when the diagnosis is in question, and it is required for species identification and molecular drug resistance testing. Most published reports have used species-specific primer–target pairs, including *M. leprae*-specific 16S ribosomal RNA (rRNA),^14^^,^^15^ the *M. leprae*-specific repetitive element (RLEP),^12^^,^^16^ or a combined *M. leprae-*specific 16S rRNA and RLEP.^17^ Assays to specifically detect and differentiate *M. lepromatosis* include the *M. lepromatosis*-specific repetitive element (RLPM)^12^ and *rpoT*.^18^ Whether HD-associated mycobacteria can be routinely detected by broad-range PCR assays remains unclear.

Broad-range PCR-based assays target conserved primer-binding sites flanking highly polymorphic regions of a gene, allowing for species-level identification after species-agnostic amplification. These assays have shown incredible promise for the clinical identification of bacteria,^19^ including fastidious organisms,^20^ other nontuberculous mycobacteria (NTM),^21^^,^^22^ and fungi.^23^ The University of Washington (UW) Molecular Microbiology Division employs a broad-range multilocus PCR strategy to detect and identify species of NTM by sequencing 16S rRNA, *rpoB*, and *hsp65* amplicons. This study describes the use of these broad-range NTM PCR assays for the diagnosis of HD owing to *M. leprae* or *M. lepromatosis* at a large clinical reference laboratory in Washington state in the United States.

## MATERIALS AND METHODS

The study, which was approved by the UW Institutional Review Board (STUDY00013877), analyzed cases from 2009 to 2024 at the UW (Seattle, WA) Molecular Microbiology Clinical Laboratory. Specimens with confirmed *M. leprae* or *M. lepromatosis* diagnoses by molecular tests were included. Accepted specimen types were fresh–frozen tissue, formalin-fixed paraffin-embedded (FFPE) tissue, and nonblood body fluids. From submitted documentation, we obtained the patient’s sex and age as well as the specimen’s originating location, order/result date, tissue type, and anatomic location. For patients seen clinically within the UW medical system, we obtained country of origin, year of immigration (if applicable), travel history, clinical leprosy characteristics (e.g., Ridley–Jopling classification), and histopathologic findings.

### Molecular methods.

All clinical testing was performed at the UW Molecular Microbiology Laboratory pursuant to its high-complexity Clinical Laboratory Improvements Amendments (CLIA) license and College of American Pathologists accreditation. Laboratory-developed processes were validated in accordance with the standards set by the Clinical Laboratory Standards Institute and monitored for quality through regular proficiency testing, biannual external inspections by the College of American Pathologists, and incorporation of control reactions as prescribed by CLIA.

DNA was extracted from both fresh and FFPE tissue as previously described^23^ except that we also validated and used multiple DNA extraction kits (Qiagen, Venlo, The Netherlands) in 2021 because of supply chain disruptions related to the coronavirus disease 2019 pandemic. Each assay used approximately 8 mm3 of FFPE tissue. Broad-range, multilocus NTM PCR included amplification of *rpoB*,^19^ 16S rRNA, and *hsp65*. Starting in 2019, the v.1–v.2 hypervariable region of the bacterial 16S rRNA gene was amplified per the broad-range bacterial (BCT) PCR protocol as previously described.^20^ Before 2019, the reverse primer was AFBsp, 5′-GCTGCTGGCACGTAGGTG-3′. The first round of the nested *hsp65* reaction was performed as described.^19^ The second-round PCR was performed using proprietary primers and probes developed and validated by the clinical laboratory.

Polymerase chain reaction amplicons, which were ∼350 base pairs (bp) for 16S and rpoB loci and ∼450 bp for the hsp65 locus, were Sanger sequenced and analyzed using Basic Local Alignment Search Tool^24^ against National Center for Biotechnology Information public databases and a curated database containing type strains and RefSeq (https://www.ncbi.nlm.nih.gov/refseq/) records.^23^^,^^25^ When sequencing results of the BCT PCR 16S products suggested mixed DNA templates, we performed amplicon-based Next-Generation Sequencing (NGS) of the v.1–v.2 16S rRNA locus (NGS-16S) reflexively by using an Illumina MiSeq instrument (Illumina, San Diego, CA) and 250-bp paired-end reads.^25^^,^^26^ Each case was reviewed by two independent certified medical laboratory scientists and a board-certified director.^23^ Turnaround time was the elapsed time between order placement and finalized molecular result.

### Sequence analysis.

Available clinical sequences were analyzed retrospectively. Primer sequences were trimmed and formed into species-specific multisequence alignments with Ugene v. 51.0^27^ using the MUltiple Sequence Comparison by Log-Expectation algorithm for each target. Wild-type reference sequences were obtained from GenBank: 16S: NC_002677 (*M. leprae*) and EU203590 (*M. lepromatosis*); *hsp65*: M14341 (*M. leprae*) and EU203593 (*M. lepromatosis*); and *rpoB*: Z14314.1:4106–7645 (*M. leprae*) and EU203594 (*M. lepromatosis*). Nonwild-type polymorphisms within 15 bases of the primer binding sites were excluded as potential PCR/sequencing artifacts. Sequences for *rpoB* covering positions 451–470 were analyzed for mutations reported to confer rifampin resistance.

## STATISTICAL ANALYSES

We used the Fisher exact test to compare categorical distributions between the species. For positive percentage agreement between NTM PCR and BCT PCR, the analysis was restricted to those samples with a positive identification by NTM PCR and concurrent BCT PCR analysis. All data analysis was performed in R software v. 4.4.3 (R Foundation, Vienna, Austria).^28^^,^^29^

## RESULTS

We identified 137 patients with 148 samples from July 2009 to December 2024 who had molecular evidence of infection with *M. leprae* or *M. lepromatosis*. *Mycobacterium lepromatosis* was identified in 10 samples (6.8%) from 8 (5.8%) patients, and *M. leprae* was identified in the remaining 138 samples (93.2%) from 129 patients. We did not detect any samples with dual infection.

Most samples (*n* = 110/148, 74%) were from 2019 or later (Supplemental Figure 1). No patient had more than two samples submitted; for the 11 patients with two samples submitted, almost all had near-simultaneous result time stamps, with the longest difference being 49 days. The median age of patients was 51 years old (range: 11–91), and it did not differ by the infecting mycobacterial species. A bimodal distribution of ages of patients infected with *M. leprae* was observed, with peaks between 30–40 and 60–70 years old (Supplemental Figure 2). Most patients (69%) were male. Of the 130 samples for which fixation information was available, 100 (77%) were FFPE, and the rest were fresh (Table 1).

**Table 1:** *Mycobacterium lepromatosis* cases are a minority of Hansen’s disease cases

Characteristic	*Mycobacterium leprae*	*Mycobacterium lepromatosis*	Total
Patients (%)	129	8 (5.8)	137
Age (years), median (range)	51 (11–90)	53 (34–91)	51 (11–91)
Sex (%), F/M/U*	29.5/69/1.5	37.5/62.5/0	30/69/1
Samples (%)	138	10 (6.8)	148
No. of states^†^	25	7	26
FFPE (%)^‡^	92/122 (75)	8/8 (100)	100/130 (77)
Sequences (%)	230	16 (6.5)	246

F = female; FFPE = formalin fixed paraffin embedded; M = male; U = unknown. Patient characteristics and limited biopsy findings are indicated for all molecular microbiology cases from July 2009 to December 2024 in which *Mycobacterium leprae* or *Mycobacterium lepromatosis* was identified. The single case of *Mycobacterium lepraemurium* is not represented in this table.

* Percentage of patients with reported sex as F, M, or U.

^†^ Originating location was available for 146 of 148 samples (98.6%).

^‡^ The preservation status of the samples (either fresh or FFPE) was available for 130 of 148 samples (87.8%).

**Figure 1.**
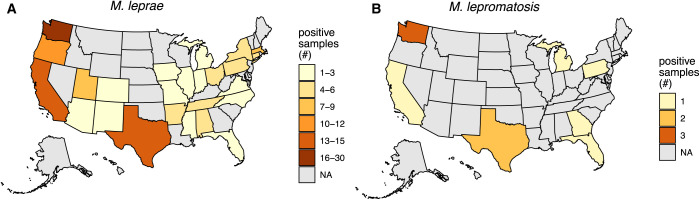
Both *Mycobacterium leprae* cases and *Mycobacterium lepromatosis* cases are geographically dispersed. Counts are by state of origin for samples positive for (**A**) *M. leprae* or (**B**) *M. lepromatosis*. (**A**) One hundred thirty-six of 138 samples (98.5%) with *M. leprae* had an originating location. One sample originating from Saudi Arabia with *M. leprae* is not shown. (**B**) Ten of 10 samples (100%) with *M. lepromatosis* had a state of origin available. NA = not applicable.

Of the 146 samples for which we had the originating location, all but 1 sample came from the United States; the exception was sent from Saudi Arabia and identified as *M. leprae*. The U.S. samples were submitted from 26 states (*M. leprae* from 25 states and *M. lepromatosis* from 7 states) (Figure 1). Washington state sent the most samples, with 25 *M. leprae-*positive samples and 3 *M. lepromatosis*-positive samples. Texas, California, and Oregon sent the next highest numbers of samples, with 14, 13, and 12 *M. leprae*-positive samples submitted, respectively (Figure 1).

**Figure 2.**
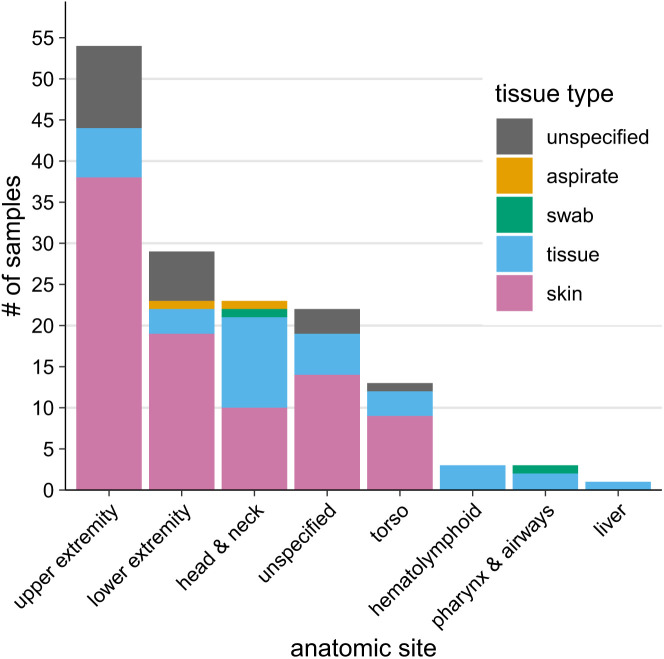
Most samples are skin biopsies from the extremities, but atypical sites are represented in a significant subset. Information for the tissue type and anatomic site for all 148 samples was received.

Although most samples were skin biopsies from the extremities, several anatomic locations not typically implicated in HD were also represented. Of the 128 samples with a known tissue type, 90 samples (70.3%) were skin, and an additional 34 samples (26.6%) were designated “tissue,” the majority of which came from the extremities and are likely skin biopsies (Figure 2); it can be conservatively estimated that ∼90% of samples were skin. Of the 126 samples with a known anatomic site, the upper extremities accounted for 54 samples (42.9%), and the lower extremities accounted for an additional 29 samples (23.0%). Samples from the head and torso comprised most of the remaining samples (*n* = 35, 27.8%). Although the number of *M. lepromatosis* cases with known anatomic site was small (10 cases), all were from the extremities (lower extremities more frequently than upper extremities) (Supplemental Figure 3), and *M. lepromatosis* was significantly more likely to be found on the extremities compared with a nonextremity site than *M. leprae* by the Fisher exact test (*P* = 0.015).

Surprisingly, 10 samples (7.9% of the 126 samples with a known site) with *M. leprae* originated from atypical anatomic sites. The head and neck included two samples (1.6%) from the eye (including an aqueous humor aspirate and a medial scleral/limbal conjunctival tumor) and one sample from the anterior tongue. Two samples were from the pharynx in addition to an unspecified “airway” biopsy. Three samples came from hematolymphoid organs, including a bone marrow biopsy and two lymph node biopsies. Finally, one sample came from a liver lesion in a patient that did not have any concurrent skin lesions (Figure 2).

Additional clinical information was available for 20 patients seen within the UW system, primarily at the Hansen’s Disease Clinic at Harborview Medical Center (Table 2). *Mycobacterium leprae* was detected in 19 patients, and *M. lepromatosis* was detected in 1 patient. Most patients (55%) were originally from Oceania, with the remaining patients from North America (20%), Asia (20%), and Africa (5%). For the 16 patients for whom the time between immigration and biopsy was available, the median was 6 years (range: 0–17). Most patients were classified as multibacillary (95%) by WHO classification and lepromatous leprosy (70.6%) by Ridley–Jopling classification. Of the 19 patients with data on histopathologic examination of either the specimen tested or a concurrent biopsy, all had histiocytic inflammation, 74% had well-formed granulomas, and 42% showed neutrophilic inflammation. Importantly, two samples (11%) were negative by AFB or Fite special stains, and the rest were positive (Table 2).

**Table 2:** Most patients in the University of Washington system are foreign born with multibacillary disease (*n* = 20)

Category	Patients (*n*)	Percentage
Region of origin		
Oceania	11	55.0
North America*	4	20.0
Asia	4	20.0
Africa	1	5.0
Years between immigration and result^†^	Median: 6, range: 0–17	
Infecting species		
*Mycobacterium leprae*	19	95.0
*Mycobacterium lepromatosis*	1	5.0
WHO classification		
Multibacillary	19	95.0
Paucibacillary	1	5.0
Ridley–Jopling classification^‡^		
LL	12	70.6
BL	5	29.4
H&E inflammation^§^		
Histiocytic	19	100.0
Granulomatous	14	74.0
Neutrophilic	8	42.0
AFB/Fite stains^¶^		
Positive	17	89.0
Negative	2	11.0

AFB = acid fast bacillus; BL = borderline lepromatous leprosy; H&E = hematoxylin and eosin; LL = lepromatous leprosy. Patients are from within the University of Washington system with additional clinical information.

* Including one patient from the United States without international travel.

^†^ Nineteen of 20 patients (95%) were born outside of the United States, and the elapsed time between immigration and molecular result was available for 16 patients.

^‡^ Ridley–Jopling classification was unknown in three patients.

^§^ H&E inflammation for samples taken on the same date of biopsy was available for 19 patients (nonexclusive).

^¶^ Fite stains were done on 16 samples. The remaining three samples had AFB stains. One of the negative samples had only an AFB stain done.

One patient was originally from the United States and reported no travel outside of the Pacific Northwest United States or known contact with a case. He was diagnosed with paucibacillary HD after identification of *M. lepromatosis* on an FFPE punch biopsy that had granulomatous and neutrophilic inflammation and no detectable acid-fast forms. Given that this represents a rarely reported case of likely autochthonous acquisition of *M. lepromatosis* in the United States, additional details are presented in a companion case report.^30^ Unfortunately, travel histories for patients were not available outside of the UW cases.

Most samples had multiple molecular assays ordered. Almost all samples (*n* = 146/148 samples or 98.6%) had an order for NTM PCR. The two remaining samples were identified solely by bacterial 16S rRNA; species-level identification was possible directly from PCR-amplified sequences in one case, and reflex NGS-16S sequencing identified *M. leprae* in the other case. The next most common test was *Mycobacterium tuberculosis*-complex PCR, which was ordered on 105 samples (70.9%), followed by BCT PCR on 27 samples (18.2%) and broad-range fungal PCR^23^ on 24 samples (16.2%). Of the 27 BCT PCR orders, 6 (22.2%) were reflexed to NGS-16S sequencing because of multiple templates detected by PCR. Four of these NGS-16S cases identified *M. leprae* (identified as major abundance in one case, minor abundance in two cases, and trace abundance in one case), one case identified *M. lepromatosis* (trace abundance), and one case did not identify any mycobacterial species (although the NTM PCR was positive for *M. leprae* in the nested *hsp65* PCR). The median turnaround time from order to result for all assays (including those that reflexed to NGS-16S) was 5.1 calendar days, with 85% of samples having a turnaround time less than 7 calendar days, including shipping time (Supplemental Figure 4).

**Figure 3.**
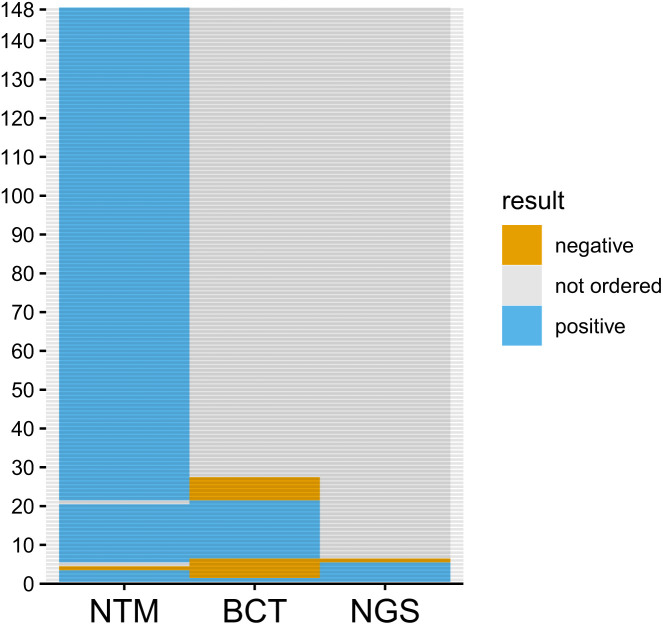
Most samples were detected by nontuberculous mycobacteria (NTM) polymerase chain reaction (PCR); broad-range bacterial (BCT) PCR has a 70.8% positive agreement with NTM PCR. Each horizontal line represents a single specimen: blue if the assay identified *Mycobacterium leprae* or *Mycobacterium lepromatosis* species, orange if the assay did not (including the presence of interfering templates), and gray if the assay was not ordered/performed. Orders for broad-range tuberculosis complex and fungal PCR assays are not shown. NGS = next-generation sequencing.

Nontuberculous mycobacteria PCR was positive in 145 of 146 cases (99.3%), with the 1 negative case detected as trace abundance *M. leprae* by reflex NGS-16S testing from a BCT PCR order. Of the 24 samples that were positive by NTM PCR and had an order for BCT PCR, 15 (62.5%) had either *M. leprae* or *M. lepromatosis* identified by BCT PCR amplification, with another 2 samples identified by NGS-16S sequencing for an overall percentage agreement of 17 of 24 (70.8%) (Figure 3).

**Figure 4.**
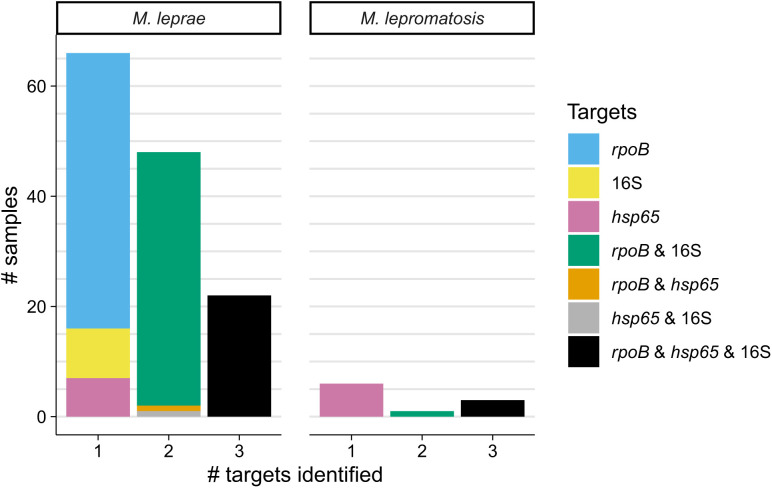
Most samples are identified by one target gene, and the distribution differs between species. The numbers of samples for which the indicated target genes were used in a positive identification for each species are grouped by the total number of targets identified. Data are shown for 146 of 148 samples. Two samples without target-level data were excluded. *M. leprae* = *Mycobacterium leprae*; *M. lepromatosis* = *Mycobacterium lepromatosis*.

Including both species, the most frequent gene target with a positive molecular result for the final species identification was *rpoB* (123 samples, 84.2%) followed by 16S (82 samples, 56.2%) and finally, by *hsp65* (40 samples, 27.4%). Half of all samples (*n* = 72/146 samples with target information, 49.3%) had species identification based off sequencing from a single gene target (Figure 4). An additional 49 samples (33.6%) were identified from two targets, and only 25 samples (17.1%) were identified from all three targets (Figure 4). This is likely in part because of fixation; fresh tissue had two targets identified in 15 of 29 samples (51.7%) and one target identified in only 9 of 29 samples (31.0%) (Supplemental Figure 5), although the distribution of number of targets identified was not significantly different (*P* = 0.09) between fixation status by the Fisher exact test.

The distribution of singly detected genes differed between the two species. For *M. leprae*, 75.8% of single-target samples (50 of 66 samples identified by a single target) were identified with *rpoB* followed by 16S (13.6%, 9 samples) and *hsp65* (10.6%, 7 samples). In contrast, for *M. lepromatosis*, all six samples identified by a single gene were identified by *hsp65* (Figure 4). Although the sample size for *M. lepromatosis* was small, these distributions are significantly different (*P* <0.001) by the Fisher exact test.

Most sequences obtained by the clinical laboratory were available for subsequent analysis. For *M. leprae*, this included 122 *rpoB* sequences, 82 16S sequences, and 32 *hsp65* sequences. For *M. lepromatosis*, four *rpoB* sequences, four 16S sequences, and eight *hsp65* sequences were available. All 16S sequences for both species had 100% nucleotide identity to reference strains as did all *M. lepromatosis hsp65* and *rpoB* sequences. Several polymorphisms of uncertain significance were identified in the *M. leprae hsp65* sequences, with all but one within 15 nucleotides of the primer binding site (Supplemental Figure 6A); raw sequencing files were not available for rereview, precluding interrogation of Phred scores. No established rifampin resistance-conferring mutations were detected. A single nonsynonymous mutation A468V in *rpoB* was detected (Supplemental Figure 6B) in a treatment-naïve patient who subsequently successfully received a three-drug regimen that included rifampin.

## DISCUSSION

Here, we report the molecular detection of the causative species of HD in 148 samples from 137 patients by a single large reference laboratory using broad-range PCR assays. Broad-range PCR assays offer several advantages over *M. leprae*-specific primers or *M. lepromatosis*-specific primers. Capitalizing on the efficiencies of a high-volume broad-range assay allowed for a turnaround time of less than 7 calendar days for the vast majority (∼85%) of assays at our center. The single-NTM assay able to detect both *M. leprae* and *M. lepromatosis* also decreases the regulatory burden of maintaining multiple high-complexity species-specific assays.

The ability to interrogate multiple NTM species simultaneously is powerful in cases with a broad differential diagnosis, especially in those where histopathology demonstrates granulomas and/or AFB and where typical clinical findings (e.g., loss of sensation or a thickened peripheral nerve) are absent or are not assessed. Specifically, we believe that implementing broad-range NTM assays will help reduce underdiagnosis of HD disease, particularly in places (like the United States) where new cases are rare, clinical awareness of HD is often low, and there is increasing evidence of autochthonous transmission (e.g., in the southeastern,^31^^–^^35^ midwestern,^36^ and northwestern^30^ United States). Furthermore, our cohort of UW patients underscores that the histopathology is often variable; only 74% had granulomas on hematoxylin and eosin stain, and 10% had negative AFB stains, suggesting that broad-range assays can be beneficial even without typical histopathologic findings.

Unexpectedly, the detection of HD-associated mycobacteria in samples from atypical locations is another benefit of broad-range assays. Although most samples (∼90%) were skin, 8% of samples came from atypical locations where HD was likely unsuspected. Ocular involvement in HD is an often neglected form of the disease,^7^ and we detected *M. leprae* in multiple eye specimens. Although the role for respiratory/aerosol spread of HD remains contentious, clinicians often submit nasal swabs for AFB smears along with slit-skin smear analysis for surveillance. We detected *M. leprae* in four oral and respiratory samples, suggesting that molecular diagnostics might facilitate further investigation of HD transmission and allow for easier clinical screening/surveillance. Our identification of *M. leprae* in bone marrow and lymphoid tissue corroborates previous case reports of *M. leprae* involving hematolymphoid organs, causing fevers and pancytopenia,^37^^,^^38^ and it warrants further investigation to understand the true rate of systemic involvement. The detection of *M. leprae* in a liver biopsy further underscores the power of broad-range assays to detect unsuspected species and raise additional avenues for research. Interestingly, we found that *M. lepromatosis* was more likely to be identified from the extremities compared with *M. leprae*, agreeing with a similar report looking at cases from Mexico.^39^

The true prevalence of *M. lepromatosis* in the United States remains unknown; previous case series have suggested its proportion among all HD cases to be as high as 15–20%,^9^^,^^11^^,^^12^ but these findings were from regions close to Mexico, where *M. lepromatosis* is known to cause almost half of HD cases.^11^ In our series, *M. lepromatosis* was detected in only 5.8% of patients. The difference is likely explained by different populations; a recent study looking at all reported HD cases in Washington state from 2001 to 2023 found that 70% of cases were among persons born in Oceania or Asia,^4^ areas with very low incidence of *M. lepromatosis*.^11^ Interestingly, one case of *M. lepromatosis* in this study was born in the United States and had no known travel to endemic regions; this case is reported in detail in a companion report.^30^ This case highlights the salience of two recent reports demonstrating the presence of *M. lepromatosis* before European contact throughout the Americas^40^^,^^41^ and a case report of likely local acquisition of *M. lepromatosis* in the Upper Midwest of the United States.^36^ These reports suggest that additional research is needed to investigate autochthonous transmission of *M. lepromatosis* in the United States. Although no dual infections were detected, Sanger sequencing assays could miss events because of low organism abundance. Although the 16S rRNA NGS could resolve mixed infections, the 16S PCR amplicon was less likely to be positive, and only six qualified for NGS-16S.

Our data support the inclusion of multiple loci (16S rRNA, *rpoB*, and *hsp65*) in the broad-range NTM PCR assay to maximize sensitivity for HD-associated mycobacteria; half of all samples were identified with a single gene target, and the most identified target (*rpoB*) was only detected in 84.2% of specimens, suggesting that one sixth of cases would be missed by any single-target assay. A combination of nucleic acid fragmentation (amplified by fixation effects) and stochastic binding/amplification likely contributes to unidentified gene targets in positive cases. Furthermore, the *hsp65* detection is probe based, but the probe has mismatches to HD-associated mycobacteria, resulting in reduced signal and increased risks of false negativity in this locus, despite being nested. Our observations suggest that the *hsp65* gene target might be particularly useful in the identification of *M. lepromatosis*, but the possible biological and/or technical reasons for the observed differences in identifying gene targets between *M. leprae* and *M. lepromatosis* require further investigation. Although the BCT assay can detect HD-associated mycobacteria, 30% of cases would have been missed if that assay was used alone, supporting the importance of a multitarget NTM assay. As expected given the strong sequence conservation among HD-associated mycobacteria,^42^^,^^43^ we found very little sequence variation and did not detect any *rpoB* mutations known to cause rifampin resistance.

The first limitation of this study is the lack of clinical information for most specimens because of our role as a reference laboratory, which made it impossible to investigate clinical outcomes associated with a specific species or molecular profile. Second, a denominator for “rule out HD” cases is impossible to calculate because of this lack of clinical information and because the broad-range assays are used in the evaluation of many different clinical scenarios, precluding both evaluation of clinical sensitivity and specificity and direct comparison with other molecular assays. Third, this is a single-center retrospective analysis with submitted specimens not necessarily representative of a single region or of the country. Finally, preanalytical factors, such as freeze–thaw cycles, dehydration, or formalin fixation time, that greatly impact molecular assays were both uncontrolled and unknown.

## CONCLUSION

Broad-range molecular assays effectively detect both *M. leprae* and *M. lepromatosis* in a wide range of clinical specimens. Implementing these methods would expedite diagnosis because these organisms are not cultivatable and because HD may not be clinically considered, even in the presence of granulomatous inflammation and/or positive AFB staining. More widespread use of broad-range molecular assays will help identify cases of this neglected disease, including atypical tissue involvement, to better understand not only the true epidemiology of HD but also, the full clinical spectrum of infections with HD-associated mycobacteria.

## Supplemental Materials

10.4269/ajtmh.25-0379 Supplemental Materials
