# Supplementary material for: Identification of Mycobacterium leprae and Mycobacterium lepromatosis Cases with Broad-Range Molecular Assays at a Single Large Reference Laboratory
Source: Am J Trop Med Hyg. 2026 Apr 21;114(6):1082–8. doi: 10.4269/ajtmh.25-0379 (PMC13235586; doi:10.4269/ajtmh.25-0379)
Supplement: Supplemental Materials [file tpmd250379.SD1.pdf]

Supplemental materials for:

Identification of *Mycobacterium leprae* and *Mycobacterium lepromatosis* cases with broad-range molecular assays at a single large reference laboratory

Gregory S. Olson, Khalil Deveaux, Diana Chiller, Dhruba SenGupta, Brad T. Cookson, Jason D. Simmons, Joshua A. Lieberman

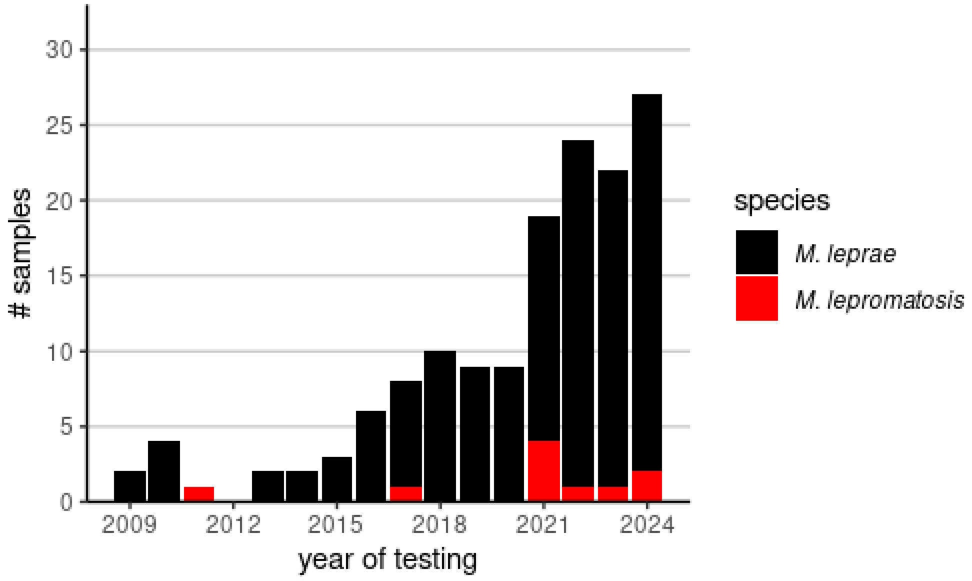

Supplemental Figure 1. The majority of positive tests have come since 2019. The number of samples by result year for which *M. leprae* (black) or *M. lepromatosis* (red) were identified.

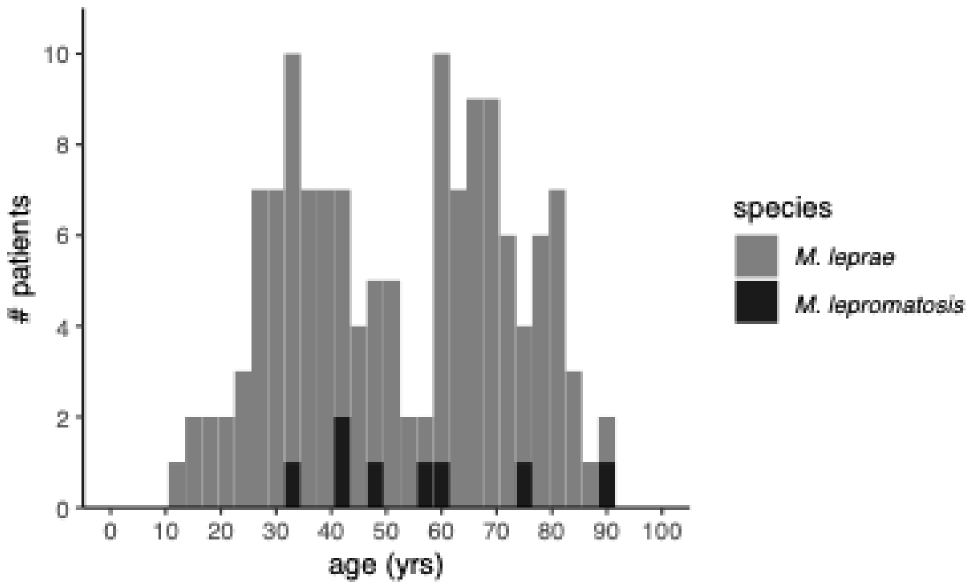

Supplemental Figure 2. Bimodal age distribution of patients with *M.leprae*. The distribution of ages by species for the 137 (100%) patients for whom we had age information.

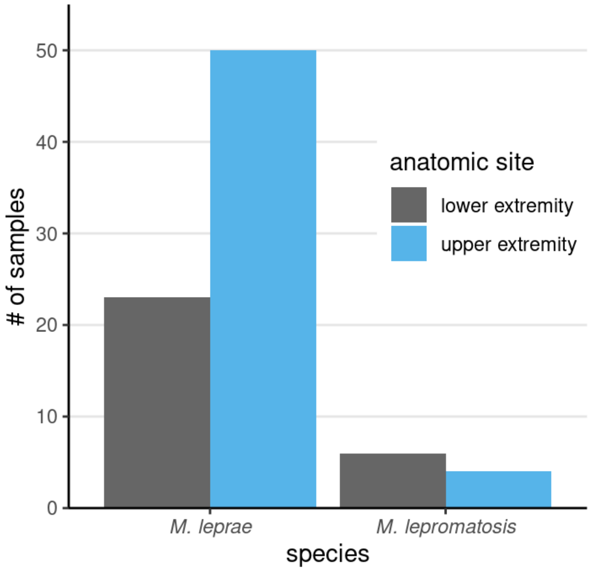

Supplemental Figure 3. Extremity sample locations for *M. leprae* and *M. lepromatosis*. All *M. lepromatosis* samples were from the extremities. Other anatomic sites for *M. leprae* are not shown.

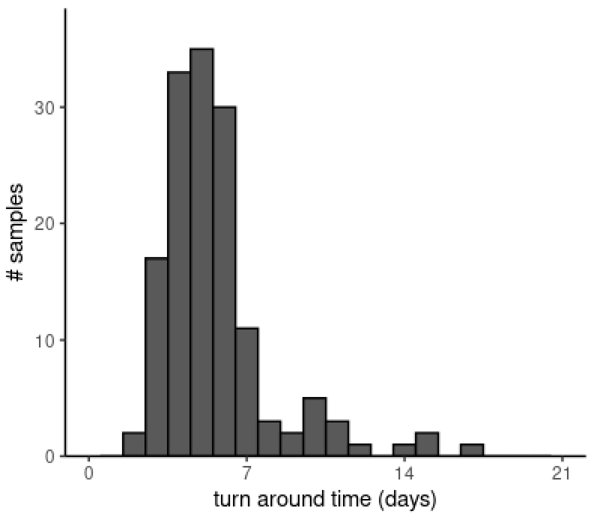

Supplemental Figure 4. Most (85%) samples have an order to result time of <7 calendar days. Turn-around time (order to result time) in calendar days for 146/148 samples. Two samples with an order to result time of <1 day were excluded. For 128/148 samples, turn-around time includes shipment of samples to the reference laboratory.

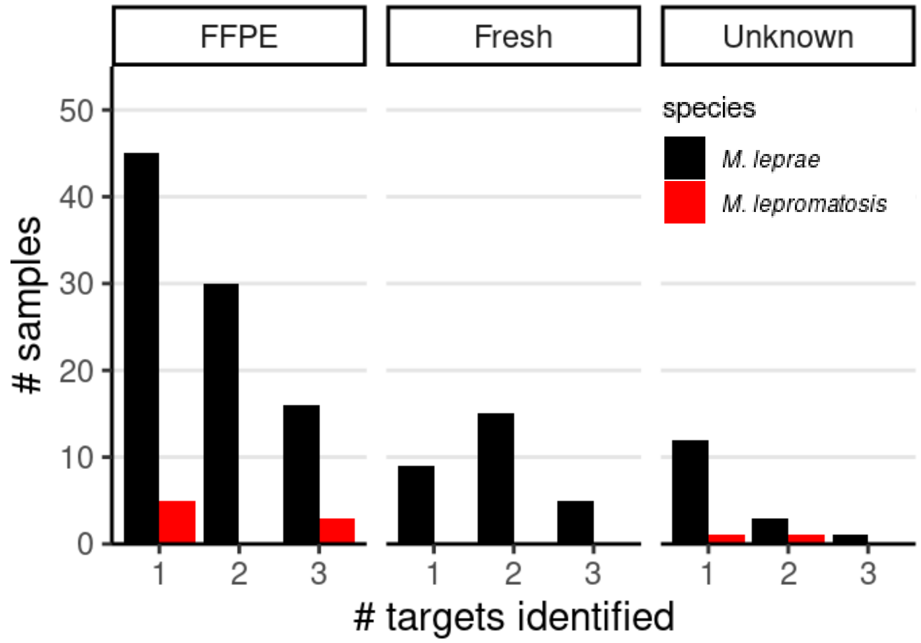

Supplemental Figure 5. The number of gene targets identified based on fixation status and species. Data shown for 146/148 samples; 2 samples without target-level data were excluded.

A

Mycobacterium leprae hps65 (GenBank Accession: M14341)

```
350-409   CACCAACGATGGCGTGTCCATCGCCAAGGAGATCGAGCTGGAGGACCCGTACGAGAAGAT
                      T
                      G
410-69    TGGCGCTGAGTTGGTCAAGGAAGTCGCCAAGAAGACAGATGACGTCGCCGGTGATGGCAC
470-529   CACGACGGCCACCGTGCTGGCCCAGGCATTGGTCAAAGAGGGCCTACGCAACGTCGCGGC
                                                 G   TTT
530-589   CGGCGCCAACCCGCTAGGTCTCAAGCGTGGCATCGAGAAAGCTGTCGATAAGGTAACTGA
                   T
590-649   GACTCTGCTCAAGGACGCTAAGGAGGTCGAAACCAAGGAACAAATTGCTGCCACTGCAGC
650-682   GATTTCGGCGGGTGACCAGTCGATCGGTGATCT
                               C
```

B

Mycobacterium leprae rpoB UW_rpoB_SV1

```
451-460 CAC AAG CGC CGG CTG TCG GCG CTG GGC CCG
       -His Lys Arg Arg Leu Ser Ala Leu Gly Pro

461-470 GGT GGT TTG TCG CGT GAG CGT GTC GGT CTT
        Gly Gly Leu Ser Arg Glu Arg Val Gly Leu-
                                  A468V
```

Supplemental Figure 6. Minimal variation observed within clinical obtained sequences. A) Scattered polymorphisms were observed in *hsp65* sequences. Polymorphisms near (< 15 nucleotides) the primer binding sites are highlighted in grey; one non-terminal polymorphism is highlighted in yellow. Raw sequencing data was no longer available for evaluation of Phred scores. B) A single non-synonymous polymorphism in the *rpoB* sequence of *M. leprae* was identified in a treatment-naïve patient resulting in a conservative amino acid change A468V. Changes in codon 468 have not been associated with rifampin resistance.
